# Wide dynamic range measurement of blood flow *in vivo* using laser speckle contrast imaging

**DOI:** 10.1117/1.JBO.29.1.016009

**Published:** 2024-01-27

**Authors:** Hong Li Liu, Yuan Yuan, Li Han, Yong Bi, Wen Yuan Yu, Yang Yu

**Affiliations:** a Beijing Anzhen Hospital of Capital Medical University, Department of Cardiovascular Surgery, Beijing, China; b Chinese Academy of Sciences, Technical Institute of Physics and Chemistry, Center of Applied Laser, Beijing, China; c University of Chinese Academy of Sciences, Beijing, China

**Keywords:** laser speckle contrast imaging, wide dynamic range, blood flow, myocardial vessel imaging

## Abstract

**Significance:**

Laser speckle contrast imaging (LSCI) is a real-time wide-field technique that is applied to visualize blood flow in biomedical applications. However, there is currently a lack of relevant research to demonstrate that it can measure velocities over a wide dynamic range (WDR), which is critical for monitoring much higher and more pulsatile blood flow in larger size myocardial vessels, such as the coronary artery bypass graft, and visualizing the spatio-temporal evolution of myocardial blood flow perfusion in cardiac surgery.

**Aim:**

We aim to demonstrate that the LSCI technique enables measuring velocities over a WDR from phantom experiments to animal experiments. In addition, LSCI is preliminarily applied to imaging myocardial blood flow distribution *in vivo* on rabbits.

**Approach:**

Phantom and animal experiments are performed to verify that the LSCI method has the ability to measure blood velocities over a wide range. Our method is also validated by transit time flow measurement, which is the gold standard for blood flow measurement in cardiac surgery.

**Results:**

Our method is demonstrated to measure the blood flow over a wide range from 0.2 to 635  mm/s. To validate the phantom results, the varying blood flow rate from 0 to 320  mm/s is detected in the rat carotid artery. Additionally, our technique also obtains blood flow maps of different myocardial vessels, such as superficial large/small veins, veins surrounded by fat, and myocardial deeper arteriole.

**Conclusions:**

Our study has the potential to visualize the spatio-temporal evolution of myocardial perfusion in coronary artery bypass grafting, which would be of great benefit for future research in the life sciences and clinical medicine.

## 1 Introduction

Visualization of myocardial blood flow varying on spatial and temporal scales is crucial for studying the pathogenesis of coronary heart disease. In particular, for the quality control of surgeries, such as coronary artery bypass grafting, there is an urgent need to constantly image the spatio-temporal evolution of myocardial blood flow perfusion.^1^^–^^3^ Currently, the transit time flow measurement (TTFM) and positron emission tomography-computed tomography are usually adopted to monitor the myocardial blood flow, but both either fail in sufficient spatial sampling or cannot perform dynamic real-time imaging. Laser speckle contrast imaging (LSCI) is an optical technique that quantifies the significant correlation between the characteristics of laser speckle and the flow rate of the light-scattering particles (red blood cells), defined as the contrast-to-blood flow relationship. Thanks to the high spatio-temporal resolution and full-field view, LSCI has attracted widespread attention in the academic community.^4^^–^^9^ The research work currently focuses on imaging blood flow in the retina, skin, and cerebral cortex and has been reported in Refs. 10–14. In addition, a handheld LSCI device was developed, bringing convenience for both patients and clinical staff.^15^^–^^18^

However, unlike the arteries in the retina, skin, and cerebral cortex,^19^^,^^20^ the coronary artery is much larger, and its blood flow is much faster and pulsatile because the heart works as a pump, performing the alternating activities of contraction and relaxation to provide energy for the blood circulation.^21^^,^^22^ Thus, an LSCI technique that meets the requirements for myocardial perfusion measurements that can measure the blood flow rate over a wide dynamic range (WDR) is a fundamental issue. As is known, the foundation of LSCI is the link between the speckle contrast (SC) and dynamics of light scattering particles. The mathematics describing this relationship depends on several factors: (1) the field correlation function $g^{1}(\tau )$, the form of which is defined by the scattering regime (single or multiple) and the motion of the particles (ordered or unordered);^5^^,^^23^^–^^26^ (2) the static scattering;^13^^,^^25^^,^^27^ and (3) the coherence parameter β.^25^^,^^26^ Based on these factors, Liu et al.^25^ derived SC models for the different cases of multiple scattering unordered motion and single scattering ordered motion and analyzed how these factors affect the accuracy of blood flow estimation. Nevertheless, whether the newly derived LSCI models could measure the wide blood flow rate range and be applied to the myocardial vessels has not been studied.

Guided by the above experimental results,^23^^,^^25^^,^^26^ we choose the ${\mathrm{SO}}_{n=2}$ (single scattering with ordered motion, where n=2 denotes n in the formula $g^{1}(\tau )=\exp(-{\left(\tau /\tau_{c}\right)}^{n}))$ LSCI model together with the precomputed coherent parameter β and the fraction of dynamic scattering ρ to derive the blood flow.^19^ We should notice, however, that different from the dynamic scattering ρ calculation in Ref. 19, the speckle patterns are recorded with a high frame rate to circumvent the vibration noise, which is necessary for WDR measurement of blood flow. A phantom experiment is designed to verify its ability to measure the blood flow rate over a WDR. Then, experiments on a rat are performed to monitor the blood flow changes with its carotid artery ligated. The results are validated by TTFM. Finally, we preliminarily apply the WDR-LSCI method to myocardial blood flow imaging *in vivo* on a rabbit heart under cardiopulmonary bypass (CPB).

## 2 Methods

### 2.1 Spatial Resolution

It is assumed that the speckle image sample statistics faithfully represent the true statistics of the speckle ensemble. This can be so only if the speckle pattern is spatially sampled at or above the spatial Nyquist rate, i.e., the speckle size is at least twice the size of a pixel. Because the normalized spatial autocorrelation function $g^{2}(\Delta x)$ of the speckle image is the point spread function of the optical system, the full width at half maximum (FWHM) of $g^{2}(\Delta x)$ is taken as the speckle size, also defined as the spatial resolution.^27^^–^^31^

Experimentally, the normalized spatial autocorrelation function $g^{2}(\Delta x)$ is measured using

$$g^{2}(\Delta x)=\frac{\left\langle I(x,y)I(x+\Delta x,y)\right\rangle }{\left\langle I(x,y)\rangle \langle I(x+\Delta x,y)\right\rangle },$$ (1)

where I(x,y) and I(x+Δx,y) represent the intensity at positions (x,y) and (x+Δx,y) of the speckle pattern. The value of the FWHM/speckle size is obtained through a Gaussian fit.

### 2.2 Choosing an LSCI Model

LSCI quantifies the SC and blood flow relationship through the intensity autocorrelation function $g^{2}(\tau )$. Briefly, the SC K is given as

$$K=\frac{\sigma_{I}}{\overset{¯}{I}},$$ (2)

where $\sigma_{I}$ and $\overset{¯}{I}$ are the standard deviation and mean intensity, respectively, over the pixels in the region of interest (ROI). Because we focus on larger blood vessels (>110  μm), we choose the single scattering with ordered motion ${\mathrm{SO}}_{n=2}$ of the field correlation function to derive the blood flow characterized by the blood flow index (BFI), defined as $x=T/\tau_{c}$, where T is the exposure time and $\tau_{c}$ is the decorrelation time.^26^ This BFI is calculated from the SC $K_{t}$, which can be determined experimentally and is given as^25^^,^^26^

$$K_{t}=\beta^{0.5}{\left\{\rho^{2}\frac{e^{-2x^{2}}+\sqrt{2\pi }erf(\sqrt{2}x)x-1}{2x^{2}}+2\rho (1-\rho )\frac{e^{-x^{2}}+\sqrt{\pi }erf(x)x-1}{x^{2}}\right\}}^{0.5}.$$ (3)

Here, erf(x) is the error function and is defined as

$$erf(z)=\frac{2}{\sqrt{\pi }}\int_{0}^{z}e^{-t^{2}}dt,$$ (4)

and $\rho =\frac{\left\langle I_{f}\right\rangle }{\left\langle I_{f}\rangle +\langle I_{s}\right\rangle }$ is the ratio of dynamic scattering, where $\left\langle I_{s}\rangle =\langle E_{s}E_{s}^{*}\right\rangle$ is the time-averaged intensity of the statically scattered light, $I_{f}=\langle E_{f}E_{f}^{*}\rangle$ is the time-averaged intensity of the fluctuating dynamically scattered light, $E_{s}$ is the static light field, $E_{f}$ is the dynamically scattered light field, and β is a normalization factor depending on the speckle size, pixel size, polarization, and coherence effects.

### 2.3 BFI Calculation

#### 2.3.1 Parameter β

The value of β is determined by the experimental setup and is typically assumed to be homogeneous across the field of view. It is a normalization factor that accounts for speckle averaging due to the speckle size and detector size mismatch, polarization, and coherence effects. The spatial speckle model, given in Eq. (5), shows that, when x tends to zero, the spatial speckle variance K will tend to $\sqrt{\beta }$. Thus, β is obtained by computing the local spatial speckle variance in the corresponding spatial windows with a slow enough flow rate and short enough exposure time. When the speckle patterns that meet the above demands are recorded, we first calculate the SC K with Eq. (2) on 7×7  pixel sliding windows and derive a SC matrix. Then, a K value is obtained by averaging the SC matrix. To improve the signal-to-noise ratio, 100 speckle patterns were chosen for each process. Finally, β was obtained through ${\overset{¯}{\overset{n}{\underset{1}{\sum }}K_{n}}}^{2}$ (n=100). The spatial SC given by the ${\mathrm{SO}}_{n\;=\;2}$ spatial LSCI model is^19^^,^^25^^,^^26^

$$K_{s}=\beta^{0.5}\{\rho^{2}\frac{e^{-2x^{2}}+\sqrt{2\pi }erf(\sqrt{2}x)x-1}{2x^{2}}+2\rho (1-\rho )\frac{e^{-x^{2}}+\sqrt{\pi }erf(x)x-1}{x^{2}}+(1-\rho {)}^{2}{\}}^{0.5}.$$ (5)

#### 2.3.2 Ratio of the dynamic scattering ρ

Typically, the speckle patterns are composed of both dynamic and stationary speckles. Although the intensity of the stationary speckles does not fluctuate in the time domain, their spatial distribution introduces a nonergodic spatial variance when the spatial SC is calculated. The statistically scattered light from tissues seriously degrades the accuracy of the flow rate estimation. Combining Eqs. (3) and (5), the ratio of the dynamic scattered photons is calculated as

$$\rho =1-\sqrt{\frac{K_{s}^{2}-K_{t}^{2}}{\beta }}.$$ (6)

#### 2.3.3 Blood flow index

**Fig. 1:**
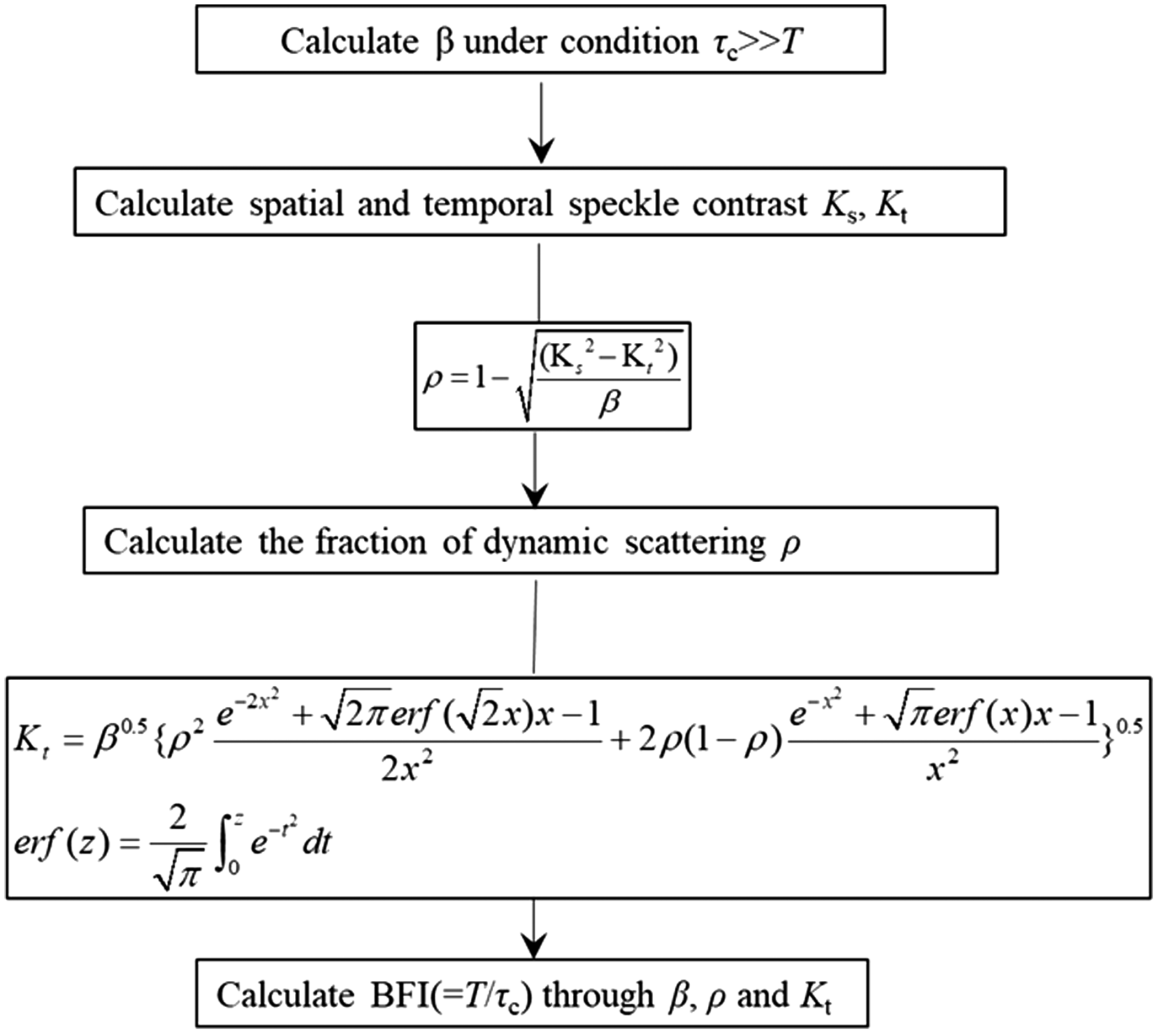
Calculation of BFI ($T/\tau_{c}$).

The procedure for deriving the BFI is shown in Fig. 1. First, the system parameter β and the proportions of the dynamic scattering ρ are derived. Second, we obtain a series of SC values K of the speckle patterns scattered from different blood flow rates. Then, we calculate the BFI ($T/\tau_{c}$) from Eq. (3). Additionally, it is essential that the speckle patterns are recorded with a high frame rate.

### 2.4 Transit Time Flow Measurement

The TTFM (Transonic, TS 420)) is used as a reference method to measure the blood flow rate. The blood flow volume detected through TTFM is acquired and saved with a National Instrument (NI) data acquisition card together with the customized LabVIEW program. TTFM is based on the fact that the time required for ultrasound to pass through blood is slightly longer upstream ($t_{u}$) than downstream ($t_{d}$). The flow volume Q is

$$Q=\mathrm{Cons}\times (t_{u}-t_{d}),$$ (7)

where Cons is automeasured by the TTFM device. Two ultrasound transducers fire ultrasound pulses in opposite directions through the bloodstream. Both pulses travel the same distance, but due to the bloodstream, the time from transmission to when the pulse is received will be different. The difference in transit time is recorded by the system and is directly proportional to the blood volume flowing through the measurement area (mL/min).

### 2.5 Animal Sample Preparation

All experimental procedures were approved by the Experimental Animal Management Ordinance of Beijing, China, and carried out in accordance with the guidelines for the humane care of animals. After shaving and disinfecting the ventral surface of the neck with iodine, the rat was placed on a heating pad. The common carotid arteries were accessed via a midline prelaryngeal incision and cleanly dissected from the sympathetic and vagus nerves. The rat was anesthetized by inhalation of 2% to 3% isoflurane in oxygen through a nose cone. The hair on the thorax was shaved, and the animal was fixed on the experimental platform. A CPB model for a rabbit with cardioplegic arrest using aortic cross clamping and cold crystalloid cardioplegia was established to remove the noise from the dynamic speckle. The sternum was divided in midline, the thymus was retracted, the aorta was isolated, and a Prolene 6-0 purse-string suture was placed in the right atrium. After systemic anticoagulation with heparin (300  IU/kg given intravenously), the proximal aortic cannulated with an 18G trocar. A venous drainage tube (inner diameter 4 mm) was placed in the right atrium. The aortic and right atrial cannulas were connected to the perfusion circuit. The distal aorta was ligated with an aortic cross-clamp and cold (4°C) cardioplegic solution infused into the aortic root.

## 3 Experimental Setup

**Fig. 2:**
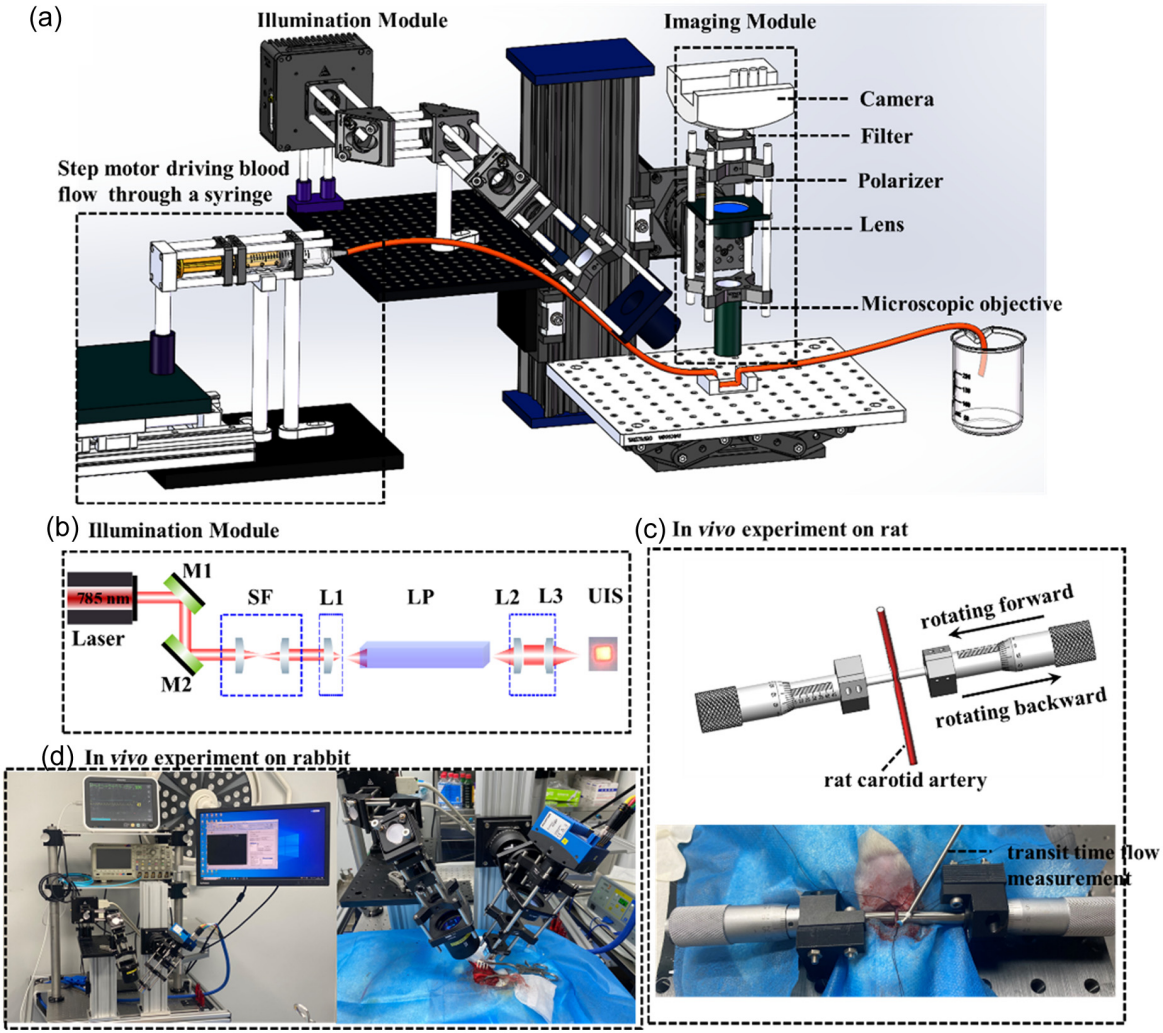
(a) Schematic of the experimental setup, including (b) the illumination module: a laser diode of wavelength 785 nm (L785H1, Thorlabs) was collimated and filtered by M1, M2, and SF. Then the laser beam was homogenized by a LP and illuminated on the sample; imaging module, consisting of microscopic objective (Nikon CF Plan NA0.13 5×), lens (AC508-100-B-ML, Thorlabs), polarizer (LPNIRE100-B,Thorlabs), filter (FL780-10, Thorlabs), camera (Mikrotron EoSens^®^ 4CXP, 2336×1728 @ 7  μm), and step motor used to drive the blood flow rate in the phantom experiment. (c) Process of rat carotid artery ligation for verifying the LSCI technique, which enables the measurement of blood flow in wide range *in vivo*. Simultaneously, the varying blood flow rate was measured by the TTFM. (d) *In vivo* experiment on rabbit. M1/M2, mirror; SF, spatial filter; L1, lens, f1=6.24  mm; L2/L3, lens, f=75  mm; LP, light pipe (3  mm×3  mm×50  mm); UIS, uniform illuminating spot.

The experimental setup is illustrated in Fig. 2(a). A laser diode of wavelength 785 nm was placed in a mount with a thermoelectric cooling stage (LDM56, Thorlabs, United States). The laser beam was homogenized by a light pipe before illuminating the sample. Figure 2(b) shows some details of the optical system. The imaging system consisted of a high-speed camera (Mikrotron EoSens^®^ 4CXP, 2336×1728 @ 7  μm), a bandpass filter (FL780-10, Thorlabs), a polarizer (LPNIRE100-B, Thorlabs), a tube lens (f=100  mm, AC508-100-B-ML, Thorlabs, United States), and an objective lens (Nikon CF Plan NA0.13 5×). Its magnification was about 2×, and the speckle size was around 4×4 camera pixels. A high acquisition frame rate card (KAYA Instrument, Israel) was used to record the dynamic scattering light.

A phantom experiment, with a microfluidic cavity (ibidi, μ-Slide I Luer, Germany) filled with rabbit blood driven by a step motor, was employed to simulate a myocardial blood vessel at different flow rates. A syringe fixed onto the step motor was connected with the inlet of the microfluidic cavity through a silicone tube. The outlet of the cavity was drained into a container. The inner areas of the syringe and the microfluidic cavity were 254 and $2\;{\mathrm{mm}}^{2}$, respectively; thus the flow rate in the latter was 127 times that of the former. In the animal experiment, we first constructed a rat (female 250 g) carotid artery ligation using two spiral micrometers fixed onto the sample platform, as shown in Fig. 2(c). At first, the two spiral micrometers were modulated to make the two end faces adjacent to the rat carotid artery, and a 0.7 mm vascular flow probe was applied to the vessel. We rotated the spiral micrometer forward until the blood flow in the carotid artery was compressed to zero and then rotated it in the opposite direction, during which the blood flow was measured by our method and the TTFM (Transonic, TS420). Then the common carotid arteries were removed and fixed in 10% buffered formalin. After 24 h of post fixation, the arteries were dehydrated, embedded in paraffin, and cut into 6  μm long sections. Standard haematoxylin-eosin staining was performed on the sections. Next, a New Zealand white rabbit (female, 6 months old) was operated upon to construct a CPB *in vivo*, and the BFI in different regions on the myocardial surface was derived.

## 4 Results and Discussion

### 4.1 Phantom Experiment

To demonstrate that our WDR-LSCI method has the capability to measure a wide range of velocities, we designed a phantom experiment. A stack of raw speckle images of the phantom was acquired with an exposure time of 49  μs and a frame rate of 20,000 Hz. A 7×7 sliding window was used to calculate the spatial SC maps, and the temporal contrast $K_{t}$ images were calculated at each pixel over 1000 consecutive frames. The system parameter β was estimated by calculating the spatial speckle variance $K_{s}$ for 100 speckle patterns that scatter from the diffuser within 4.9 ms. The β ($=K_{s}^{2}$) of our setup was derived to be 0.48. Next, we set the blood flow in the microfluidic cavity to be zero, i.e., the red blood cells were allowed to undergo natural Brownian motion, and the speckle images were recorded. Using Eq. (6), ρ was calculated to be 0.93. With β and ρ determined, the BFI value was extracted by interpolation using the contrast-to-blood flow relationship of Eq. (3).

**Fig. 3:**
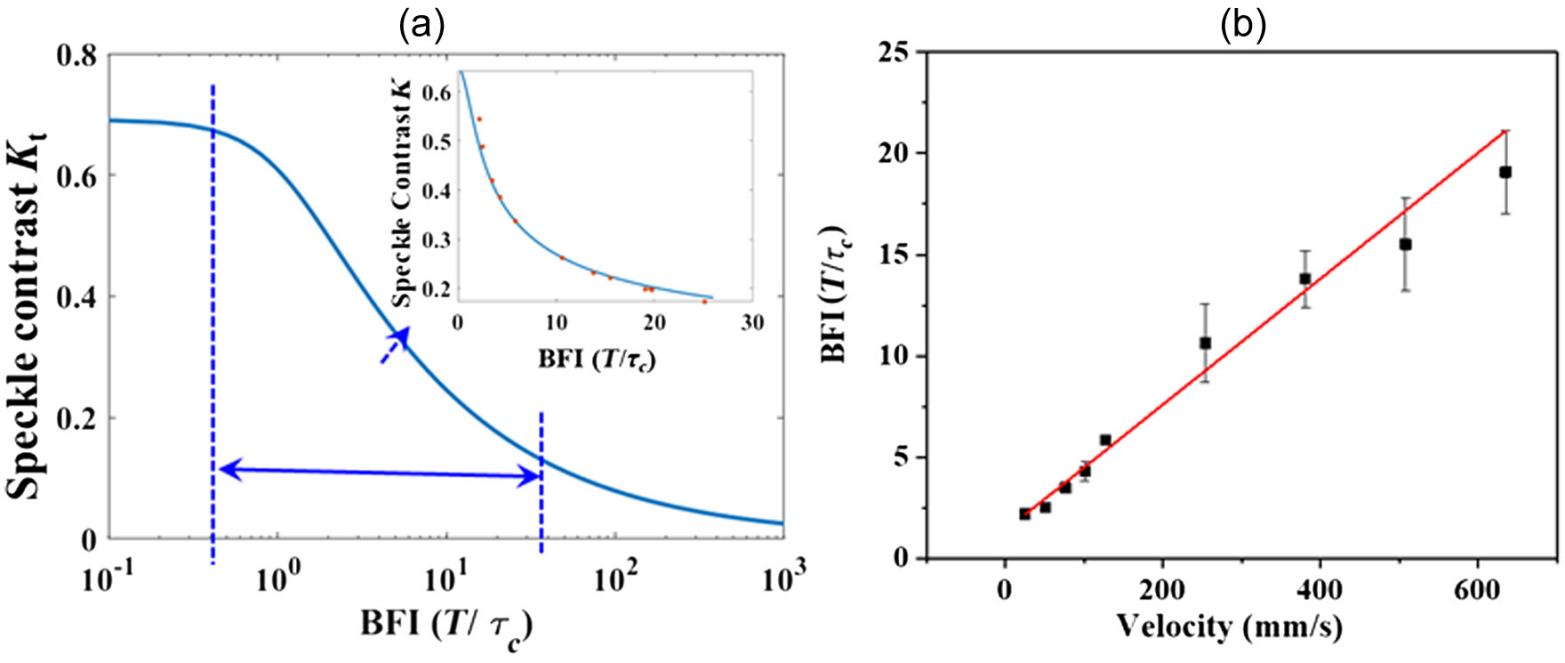
Theoretical simulation of the SC K versus the BFI ($T/\tau_{c}$) with 7% static scattering and (inset) experimental data (red dots); blue solid lines are the simulated curve. (b) The BFI versus driving blood flow rate. Square dots, experimental values; red solid line, fitted curve (BFI=1.38+0.031v).

Figure 3(a) is a theoretical simulation of the SC K versus the BFI ($T/\tau_{c}$) on a logarithmic scale with 7% static scattering. The region enclosed by the blue dashed lines is enlarged in the inset, in which the experimental data (red dots), shown on a linear scale, are the averaged values of three groups of data as the step motor was driven at varying rates from 0.2 to 8  mm/s. The corresponding flow rate in the microfluidic cavity varied from 25.4 to 635  mm/s. The values of K in the simulated K-BFI plot agree well with the experimental data. Figure 3(b) shows the linear relationship between the BFI ($T/\tau_{c}$) and the blood flow rate from 25.4 to 635  mm/s. The black squares correspond to the experimental data, and the red line is the fitted curve, plotted as BFI=1.38+0.031v, where v is the absolute blood flow (mm/s). The results indicate that β and ρ are critical parameters for deducing the BFI, and their accurate determination can greatly improve both the linearity and WDR.^13^^,^^19^^,^^25^^,^^26^ Conventionally, when it comes to realizing a WDR for a flow rate with LSCI, the multi-exposure technique in which the camera exposure time range has been clearly given has been mentioned.^13^^,^^14^ However, for obtaining the blood flow rate, compared with 15 exposure times from 50  μs to 80 ms with a standard multi-exposure speckle imaging (MESI) and 6 exposure times from 1 to 20 ms with a synthetic MESI implementation, in our LSCI setup, we only used 50 ms (for 1000 speckle images with 50  μs exposure time).

### 4.2 Animal Experiment Validation

**Fig. 4:**
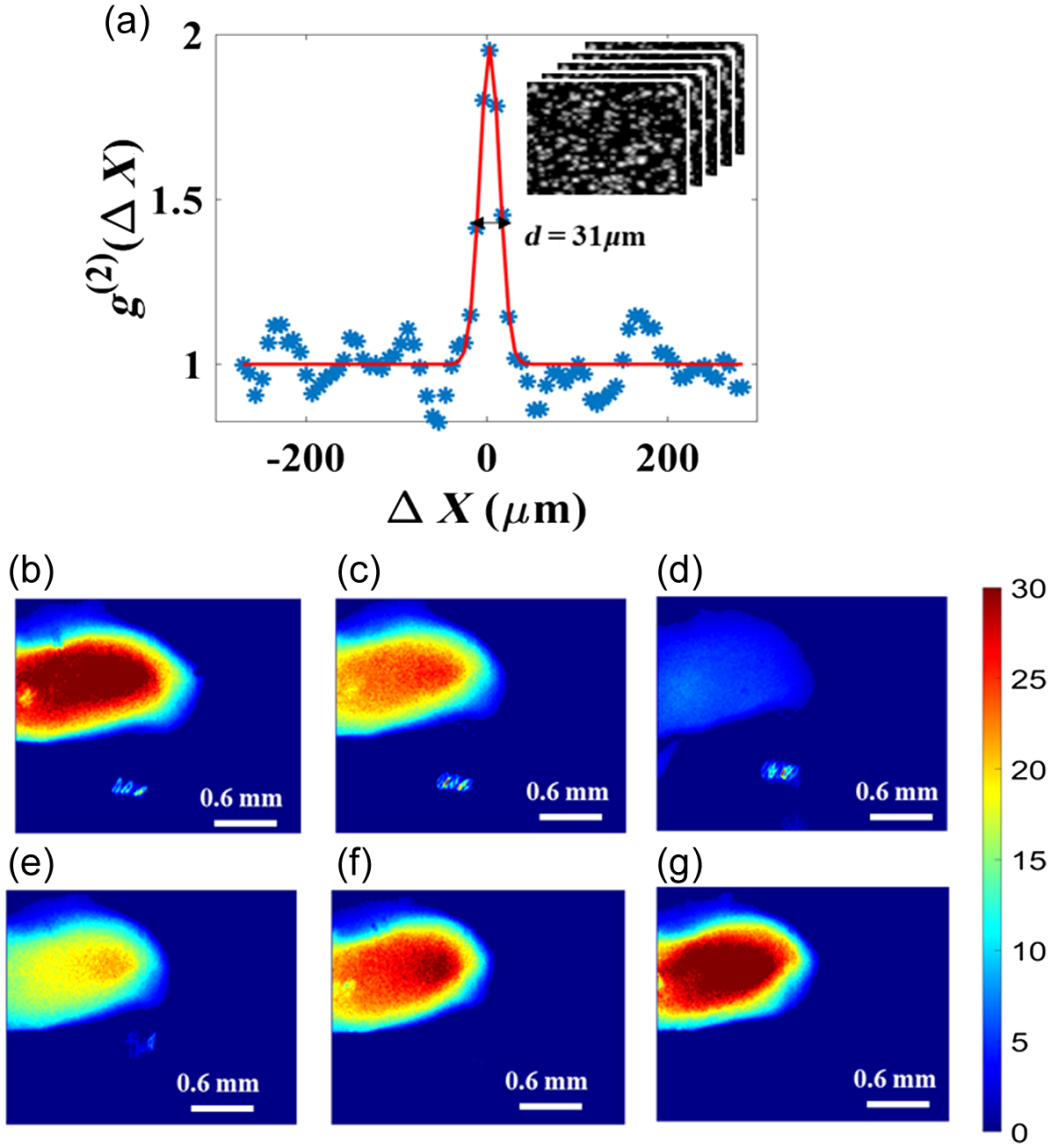
(a) Second-order autocorrelation $g^{2}(\Delta X)$ as a function of distance with 2000 measurements. Inset: the speckle patterns recorded to calculate the spatial resolution. The FWHM corresponding to the spatial resolution or speckle size is 31  μm. Blue dots, experimental data; solid curve, Gaussian fit. (b)–(g) During the process of the rat carotid artery being ligated and back to normal, its blood flow map, estimated by BFI ($T/\tau_{c}$), varies on the spatial and temporal scales at times 11, 12, 13, 14, 15, and 16 s. Color bar: red represents larger blood flow rate, and blue indicates lower flow rate.

To support the phantom results, we compared blood flow changes during carotid artery ligation estimated with both LSCI (BFI: $T/\tau_{c}$) and TTFM. Before performing the animal experiment, the normalized spatial autocorrelation function $g^{2}(\Delta x)$ versus Δx was calculated using Eq. (1), in which the intensities at a fixed pixel point (x,y) are convoluted with those of the other speckles at (x+Δx,y) for each speckle pattern. As shown in Fig. 4(a), a series of speckle images are recorded in the inset, and the 31  μm FWHM of $g^{2}(\Delta x)$ of the peak gives the resolution of the system.

**Fig. 5:**
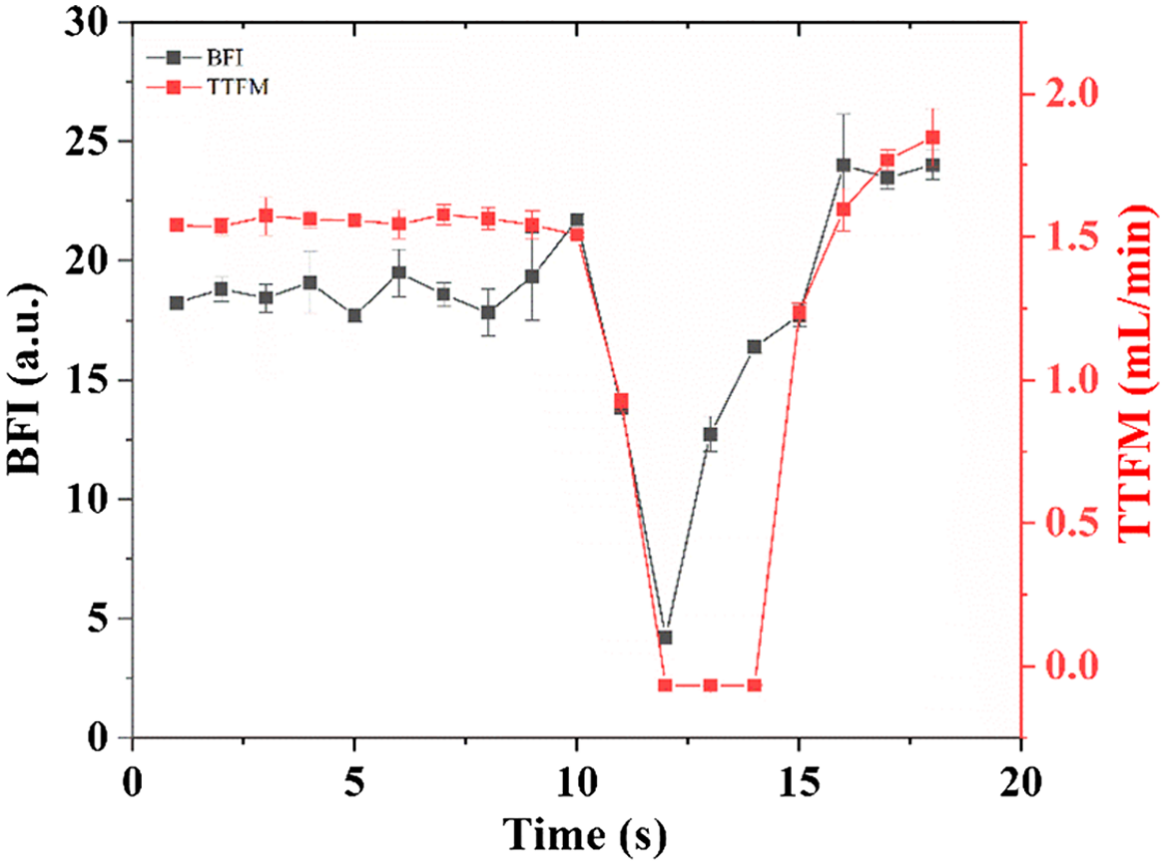
Blood flow changes during carotid artery ligation, estimated with LSCI (black dots and line) and TTFM (red dots and line).

Figure 2(c) illustrates the experimental setup that we constructed to perform a rat carotid artery ligation. The micrometer was rotated forward until the blood flow of the carotid artery was compressed to zero and then rotated backward, during which both the speckle data and the blood volume were simultaneously monitored by LSCI and TTFM. The ratio dynamic scattering from the carotid artery ρ was measured to be 0.98. From Figs. 4(b)–4(g), we can see that the blood flow rate map of the rat carotid artery characterized by BFI decreases and then becomes normal again, corresponding to the BFI values in Fig. 5 at times 11, 12, 13, 14, 15, and 16 s. The color bar value, i.e., BFI value, is deduced to range from 0 to 30. Red represents a higher blood flow, and blue indicates a lower flow rate. In Fig. 5, the black/red dots and lines represent the results measured by LSCI and TTFM, respectively. The blood flow measured with both methods at first fluctuates around a constant, then slows down to an extremely low value, and then rises. Compared with TTFM, LSCI performs better in line with the vessel unbinding process and has a higher sensitivity. During the period from 12 to 15 s when the carotid artery was gradually loosened, the BFI was 4.22, 12.73, 16.38, and 17.71, and the results measured by TTFM sharply increased, resulting in some loss of detail.

**Fig. 6:**
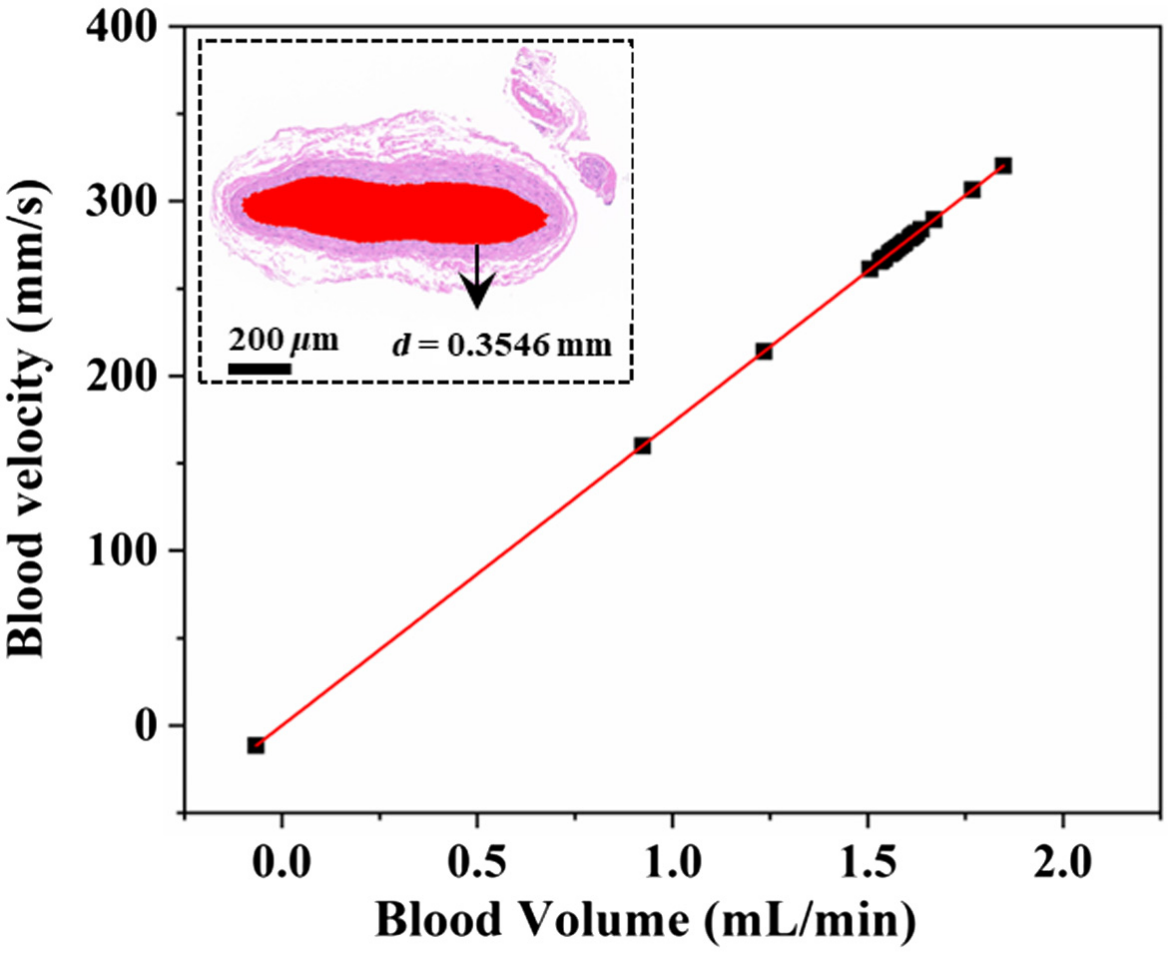
Relation between blood flow rate and blood volume in rat carotid artery. Inset: rat carotid artery slice. The averaged inner diameter is 0.3546 mm, which is measured by the software in the red area.

To further deduce the blood flow rate from TTFM, the diameter of the rat carotid artery was measured. A segment of the artery sample was fixed by 4% paraformaldehyde, then trimmed, dehydrated, embedded, sliced, dyed, and sealed. Finally, qualified samples were chosen through microscopic inspection. First, the panoramic slice scanner (3DHISTECH, Hungary, PANNORAMIC) scans and images the tissue slice. Then, the scanning software (3DHISTECH, Hungary, CaseViewer2.4) and analysis software (Media Cybemetics, United States, Image-Pro Plus 6.0) are used to measure the diameter of the blood vessel. As shown in the inset of Fig. 6, the inner diameter of the rat carotid artery (354.6  μm) is the average diameter measured in the red area by the software (Media Cybemetics, United States, Image-Pro Plus 6.0). The blood flow rate (blood volume/inner area/s) during the ligation process is shown in Fig. 6, where the blood flow volume from 0 to 1.8472  mL/min corresponds to the flow rate of 0 to 320  mm/s. Compared with TTFM, LSCI not only measures the blood flow rate over a WDR, but also has a higher sensitivity. Additionally, LSCI obtains a wide-field two-dimensional blood flow rate in a noncontact way, which can be applied in observing spatio-temporal changes of the blood flow rate in the ROI, especially in the spatio-temporal evolution of myocardial blood flow perfusion.

### 4.3 Preliminary Application of WDR-LSCI Method in Myocardial Blood Flow Imaging

**Fig. 7:**
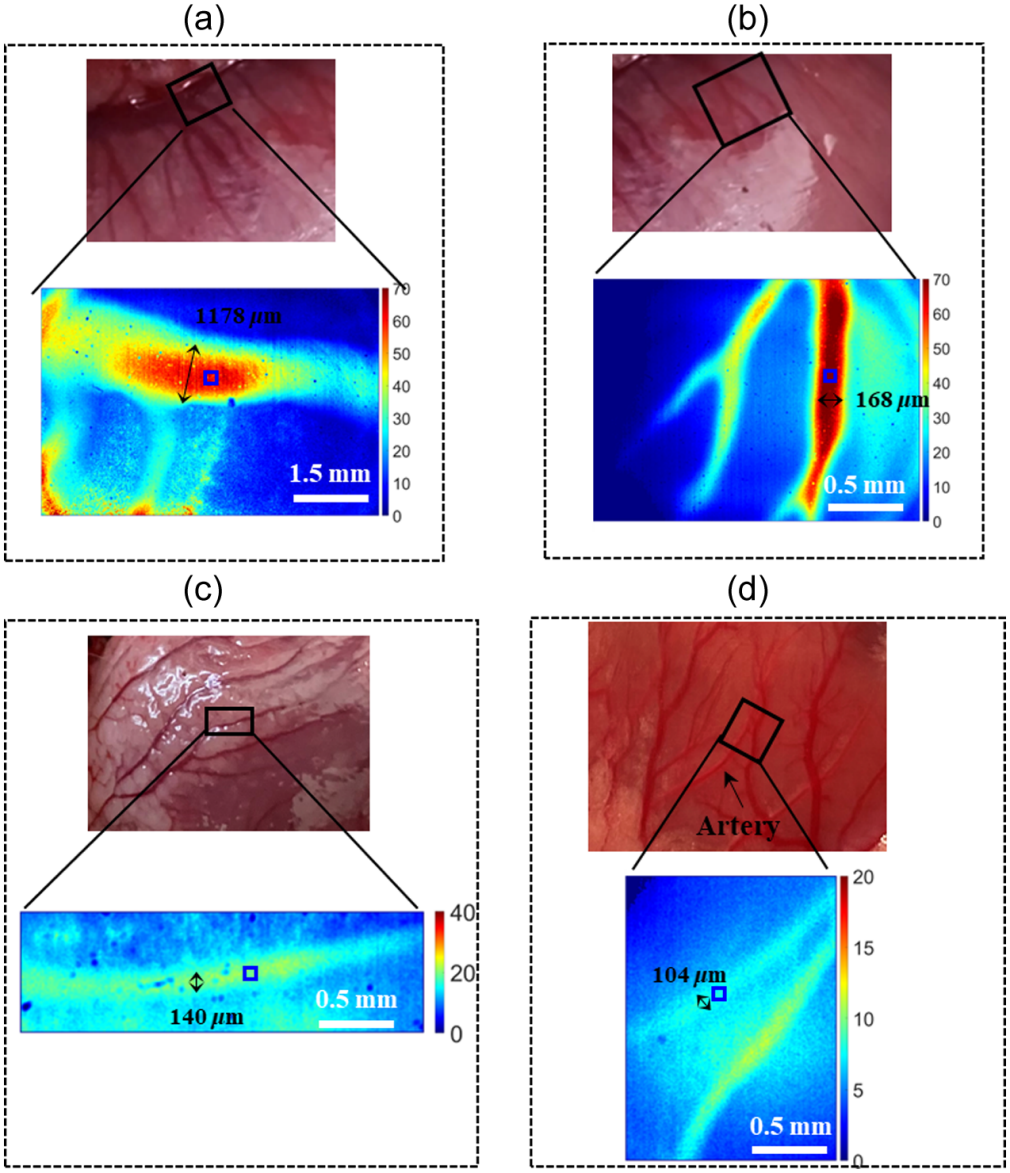
BFI ($T/\tau_{c}$) maps of different myocardial vessels corresponding to the region in the black box with individual dynamic scattering fractions ρ that are calculated in the region enclosed by a blue square: (a) large veins with a diameter of about 1178  μm and ρ=0.965; (b) small veins with a diameter of about 168  μm and ρ=0.992; (c) veins surrounded by fat with a diameter of about 140  μm and ρ=0.985; and (d) myocardial arteriole with a diameter of about 104  μm and ρ=0.971.

If an LSCI device could monitor blood flow over a wide range, its function would be greatly expanded. Specifically, it could be applied to imaging myocardial blood flow distribution, which would benefit visualization of the spatio-temporal evolution of the myocardial perfusion and provide important information for studying the pathogenesis and surgery of coronary heart diseases. Here, we preliminarily apply our WDR-LSCI method to myocardial blood flow imaging. Irregular motion artifacts from the heart beating and ventilator do significantly affect the measurement accuracy of the myocardial blood flow rate in our LSCI method. Chizari et al.^17^ proposed a model based on the optical Doppler effect to predict SC drop as an indication of motion artifacts. However, the main focus of our paper is the WDR measurable by LSCI, with the possibility of its application to myocardial perfusion imaging *in vivo*. Thus, in our experiment, we utilized a CPB model for a rabbit with cardioplegic arrest and a high-speed camera to circumvent motion artifacts. The regions of interest include large and small veins, as well as veins surrounded by fat and myocardial arteriole, the diameter of which, marked by a black arrow, is 1178, 168, 140, and 104  μm, as shown in Fig. 7. The vessels in the black box are the raw vessel images illuminated by white light and the corresponding BFI map as deduced by ${\mathrm{SO}}_{n\;=\;2}$ LSCI together with coherent parameter β (=0.48) and dynamic scattering fraction ρ. We should notice, however, that, unlike the simulation and phantom results, the ρ in the vessel of the four regions needs to be calculated separately because the optical properties of turbid media across the four regions marked by black boxes are nonergodic. We chose 20×20  pixels marked by the blue boxes to deduce ρ to be 0.965, 0.992, 0.985, and 0.971. From Fig. 7, it is clear that WDR-LSCI can provide good images of the myocardial blood flow distribution. Indeed, Padmanaban et al.^32^ found that there is a consistent change in the ρ factor as the channel diameter increases, i.e., increasing the tube diameter causes a higher drop in the SC for a certain volumetric flux. There are two reasons for the ρ values not being consistent with the results of Padmanaban et al.^32^: (1) The heart of a rabbit is small, and its surface is not flat. We have to translate or tilt the LSCI system to focus on the ROI, which leads to variation in the coherent parameter β. (ii) The optical properties of vessels in the four ROIs are all different; for example, because the scattering coefficient of the fat is larger than that of myocardial tissue, the exposure time has to be reduced when imaging the ROI in Fig. 7(c); the myocardial arteriole is located deeper, so the exposure time has to be longer. These changes would affect the value of β. In this work, we simply assume β to be invariant when calculating ρ, so the ρ values here do not increase or decrease with the vessel diameter.

## 5 Conclusion

We have verified the ability of the ${\mathrm{SO}}_{n\;=\;2}$ LSCI method in measuring blood flow in WDR from phantom experiments to animal experiments. First, the coherent parameter β was estimated by calculating local spatial speckle variance with a stationary diffuser. Second, the fraction of dynamic scattering ρ was extracted by the spatial and temporal SC. Finally, the BFI ($T/\tau_{c}$) map was obtained by the interpolation algorithm. In the phantom experiment, the full blood in the microfluidic cavity was driven by the step motor. The WDR-LSCI method was demonstrated to measure the blood flow rate up to 635  mm/s, which is the highest measurable flow rate by laser speckle method, to the best of our knowledge. To validate the phantom results, the WDR-LSCI model was applied to the rat carotid artery and compared with the TTFM, with the blood flow being controlled by two spiral micrometers. The varying blood flow rate from 0 to 320  mm/s was detected in the rat carotid artery. Furthermore, we preliminarily applied the WDR-LSCI method to myocardial blood flow imaging in the regions of interest, including larger veins, smaller veins, veins surrounded by fat, and myocardial arteriole, and clear BFI maps were obtained.

In conclusion, the WDR-LSCI method with coherent parameter β and fraction of dynamic scattering ρ was demonstrated to possess excellent performances with the following results: (1) it had the ability to measure the high blood flow rate to 635  mm/s; (2) it had a superior performance in measuring the blood flow rate compared to the gold standard (TTFM), i.e., had a higher sensitivity and could obtain a wide-field, high-precision blood flow rate map in the non-contact way; (3) it enabled imaging of the regional myocardial blood flow distribution *in vivo*. These results provide significant guidance for future biomedical studies of high spatio-temporal resolution imaging and the spatio-temporal evolution of myocardial perfusion. In future work, we intend to address the motion artifacts caused by cardiac quasi periodic motion and respiration, as well as to detect the location of the stenosis,^33^^,^^34^ which is a critical step in quantitatively monitoring the spatio-temporal evolution of myocardial perfusion in the beating heart *in vivo* before and after coronary artery bypass grafting.

## Data Availability

The data that support the findings of this article are not publicly available due to privacy. They can be requested from the author at yyuan@mail.ipc.ac.cn.
